# Diabetes-related amputations in Germany: analysis of time trend from 2015 to 2022 and differences by area-level socioeconomic deprivation

**DOI:** 10.25646/12026.2

**Published:** 2024-04-23

**Authors:** Oktay Tuncer, Yong Du, Niels Michalski, Lukas Reitzle

**Affiliations:** Robert Koch Institute, Department of Epidemiology and Health Monitoring Berlin, Germany

**Keywords:** Diabetes, Amputations, Quality of care, Diabetes-Surveillance, Social deprivation, Germany

## Abstract

**Background:**

Diabetes-related amputations reduce health-related quality of life and are an indicator of the quality of care of diabetes.

**Methods:**

Population-based age-standardized rates for diabetes-related cases of major and minor amputation were calculated and reported for the years 2015 – 2022 using the Diagnosis-related groups statistics. For 2022 these rates were also reported according to area-level socioeconomic deprivation.

**Results:**

Diabetes-related major amputations decreased from 6.8 to 5.2 per 100,000 residents in women and from 18.6 to 17.5 per 100,000 residents in men between 2015 and 2022. In 2021 and 2022, there was no further decrease in men compared to the previous year. Diabetes-related minor amputations decreased in women between 2015 and 2022, but increased in men. Amputation rates were higher in regions with high deprivation than in regions with low deprivation.

**Conclusions:**

Diabetes care should consider socioeconomic differences into account. The monitoring of the trends in amputations needs to be continued.

## 1. Introduction

Diabetes mellitus is a chronic disease characterized by elevated blood glucose levels. It increases the risk of cardiovascular disease [1] and the risk of damage to the kidneys (nephropathy) [2] and the nervous system (polyneuropathy) [3]. In combination with circulatory disorders, polyneuropathy can lead to the diabetic foot syndrome [3, 4], which can necessitate amputation in the event of complications such as uncontrollable infections or irreversible circulatory disorders [5]. Amputations lead to severe reduction of health-related quality of life and high costs for the healthcare system [4, 6].

The St Vincent Declaration of 1989, which was adopted by international experts on diabetes mellitus under the auspices of the WHO and the International Diabetes Federation, set the considerable reduction in amputation rates as a goal for improving diabetes care [7]. The National Health Care Guideline (NVL) on the therapy of type 2 diabetes recommends optimal blood glucose control and regular foot examinations to prevent secondary diseases and complications such as diabetic foot syndrome [8].

As the majority of amputations could be avoided through optimal treatment [4, 9, 10], the rate of diabetes-related lower limb amputations above the ankle (major amputations) is reported by the Organisation for Economic Co-operation and Development (OECD) as an indicator for the quality of diabetes care [11]. The Diabetes Surveillance at the Robert Koch Institute incorporated it as a core indicator [12]. The frequency of diabetes-related amputations varies greatly regionally [13] and depends heavily on the socioeconomic situation [14]. In this study, both major and minor amputations (amputations below the ankle joint) were observed over time from 2015 to 2022. In addition, the amputation rates for 2022 are stratified according to the level of socioeconomic deprivation of the region of residence.

## 2. Indicator

The study is based on data from the Diagnosis-related groups statistics (DRG statistics), which is gathered by the Federal Statistical Office of Germany and used for the cost accounting of hospital services [15]. The DRG statistics contain information on the age, gender and place of residence of the patients as well as information on illnesses and surgeries for all approximately 19 million hospital cases per year in Germany. As the DRG statistics do not contain information on income and education, the German Index of Socioeconomic Deprivation (GISD Release 2022 v0.2) [16] was used to analyze socioeconomic differences. The index comprises information on the education, employment and income situation of all districts (known as Kreise) and divides them into quintiles ranging from low to high socioeconomic deprivation [17]. Quintile 1 reflects districts with low socioeconomic deprivation, quintiles 2 to 4 those with medium deprivation and quintile 5 regions with high socioeconomic deprivation. The GISD was linked to the hospital statistics via the patient’s district of residence.

The diabetes-related amputation cases were provided by the Federal Statistical Office of Germany aggregated and stratified by year, sex, 5-year age groups and quintiles of area-level socioeconomic deprivation. The analysis included all hospital cases with a main or secondary diagnosis of diabetes (ICD-10-GM codes E10.-/E11.-/E13.-/E14.-) of persons aged 15 years and older in the years 2015 – 2022 for whom a lower limb amputation was documented during hospitalisation. A distinction was made between major (OPS codes 5-864/5-865.0) and minor amputations (OPS codes 5-865 excl. 5-865.0). Hospital cases were excluded if they were transferred from a rehabilitation facility or another acute care hospital, if they died during their stay, were discharged within 24 hours, had a main or secondary diagnosis of a bone cancer in the lower extremity (ICD code: C40.2/C40.3), or if a traumatic amputation of the lower extremity (ICD code: S78.-/S88.-/S98.-/T05.3-5/T13.6) or a treatment related to pregnancy or childbirth was documented.

The rates for diabetes-related major and minor amputations were calculated per 100,000 residents using the population of the respective reporting year according to population projection of the census [18]. The rates were age-adjusted according to 5-year age groups from 15 to 84 years and for persons over 84 years using Germany’s resident population at December 31, 2022 as standard population.

## 3. Results

**Figure 1:**
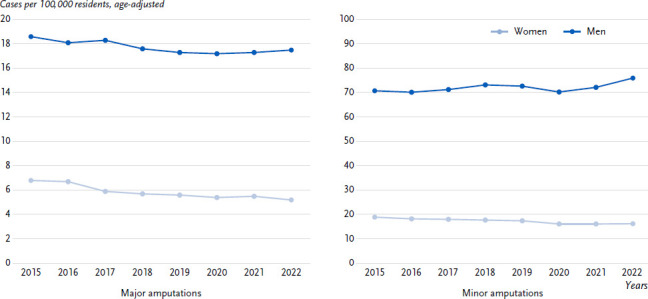
Time trends of diabetes-related major and minor amputation rates per 100,000 residents by sex. Source: hospital statistics (DRG) [15]

Both diabetes-related minor and major amputations were significantly more common in men than in women over the entire period. Between 2015 and 2022, the rates of diabetes-related major amputations in men decreased overall from 18.6 to 17.5 per 100,000 residents and in women from 6.8 to 5.2 per 100,000 residents (Figure 1), with exceptions in 2021 and 2022 in men and 2021 in women, where there was no decrease compared to the previous year. In 2022, there were 5,702 cases of diabetes-related major amputations of the lower extremity in men and 2,084 amputations in women.

The diabetes-related minor amputation rates in men rose from 70.8 cases per 100,000 residents in 2015 to 76.0 in 2022 (24,624 amputations) (Figure 1). In 2016, 2019 and 2020, there were no increases compared to the previous year. The diabetes-related minor amputations in women showed a continuous decline from 2015 (19.0 per 100,000 residents) to 2022 (16.3 amputations per 100,000 residents or 6,586 amputation cases) with the exception of 2021 and 2022.

The rates of diabetes-related major amputations were lowest in districts with low socioeconomic deprivation, with 4.1 in women and 13.3 per 100,000 residents in men (Table 1). The rates were highest in districts with high deprivation (women: 7.7; men: 22.4). The same pattern was observed for diabetes-related minor amputations. The rates were lowest in districts with low socioeconomic deprivation (women: 15.8; men: 59.8) and highest in districts with high socioeconomic deprivation (women: 21.3; men: 84.7).

## 4. Discussion

The study follows up on previous trend analyses, which reported a decrease in the rates of diabetes-related major amputations in both sexes from 2005 to 2014 and to 2015, respectively [19, 20]. According to our results, this trend continued in women until 2022; only in 2021 there was no decrease compared to the preceding year. In men, the downward trend lasted until 2020. A study using claims data of the statutory health insurance AOK showed that hospitalization rates were generally lower during the COVID-19 pandemic. At the same time, hospitalizations due to major amputations in men with diabetes did not continue to decline [21]. This is consistent with our finding that the downward trend in men has not continued since 2020 and that major amputation rates even increased slightly in 2021 and 2022. With regard to diabetes-related minor amputations, an analysis of DRG statistics observed an increase from 2005 to 2015, particularly in men [20]. In our analysis, minor amputations increased overall between 2015 and 2022. In women, minor amputations decreased from 2015 to 2020 and then stagnated. The observations raise the question of whether the optimal care of people with diabetic foot syndrome could have been upheld during the pandemic. For example, it was reported that people with diabetes received significantly less specialist care during the pandemic [22]. General practitioners also reported lower health care utilization during the lockdown in 2020 [23] and the Central Research Institute of Ambulatory Health Care in Germany (Zi) observed lower utilization of outpatient health services across all specialties corresponding to the contact restrictions [24].

Regions with high socioeconomic deprivation have higher amputation rates. Socioeconomic differences in the prevalence of diabetes may contribute to this. The differences in the prevalence may at least partially be the result of differences in behaviour-based and contextual risk factors [25]. A systematic review provides evidence that complications such as the diabetic foot, which can lead to amputations, occur more frequently in groups with low education or income [26]. Furthermore, care following the guidelines for diabetes and diabetic foot syndrome affects the risk of amputation [27]. Results from the German National Health Interview and Examination Survey 1998 and the German Health Interview and Examination Survey for Adults (2008 – 2011) show that there are no educational differences with regard to care indicators such as the annual medical examination of the foot as a whole. However, access to multi-professional treatment could differ depending on the socioeconomic situation of the residential region, as the density of medical specialists in highly deprived regions is significantly lower than in regions with low deprivation [28]. The risk of amputations could therefore be higher in regions with high socioeconomic deprivation.

## 5. Limitations

This analysis is based on the DRG statistics, which consists of case-based information. Repeated hospital visits of one person are included in the statistics as separate cases. Data quality depends on the coding practice and other documentation effects. While the documentation quality of surgical procedures such as amputations in hospitals can be regarded as high, it is possible that few non-diabetes-related amputations were included in the analysis. The calculated rates were not adjusted for diabetes prevalence. There are regional differences in the amputation rates for both sexes, which correspond to the regional distribution of diabetes prevalence. This must be considered in particular when interpreting the data according to area-level socioeconomic differences, as diabetes prevalence is higher in regions with high socioeconomic deprivation than in regions with low deprivation. Using the GISD, socioeconomic differences were analyzed with an ecological study design. This approach cannot replace a measurement of socioeconomic position at the individual level.

## 6. Conclusion

Diabetes-related amputations are an important indicator of the quality of care for people with diabetes. This study shows heterogeneous trends in amputation rates by sex and with regard to major and minor amputations since 2015. For men in particular, amputation rates have risen in 2021 and 2022. The rates are significantly higher in districts with high deprivation than in less deprived districts. Altogether, the results indicate that appropriate care for people with diabetic foot syndrome might not have optimally ensured during the COVID-19 pandemic. In particular, attention should be given to men and people in socioeconomically disadvantaged regions. The analysis of the time trend should be continued to assess future developments and socioeconomic differences should be investigated in more detail. Future analyses should also focus on possible approaches for the prevention of amputations.

**Corrigendum**

In Section 2, ‘Indicator’, on page 2, the inclusion criterion for hospital cases with diabetes was not fully reproduced; two ICD-10-GM codes (E11.- and E13.-) were missing.

The correct sentence is: ‘The analysis included all hospital cases with a main or secondary diagnosis of diabetes (ICD-10-GM codes E10.-/E11.-/E13.-/E14.-) of persons aged 15 years and older in the years 2015–2022 for whom a lower limb amputation was documented during hospitalisation.’

The article has been corrected accordingly.

## Key statement

- The rate of diabetes-related major amputations decreased in men continuously from 2015 to 2020 and remained at this level in 2021 and 2022.
- Diabetes-related amputations are more common in women and men in socioeconomically deprived regions than in regions with less deprivation.

## Figures and Tables

**Table 1:** Number of diabetes-related minor and major amputations of the lower extremity per 100,000 residents by sex, age and area-level socioeconomic deprivation 2022. Source: hospital statistics (DRG) [15]; GISD Release 2022 v0.2 [16, 17]

		Minor amputations		Major amputations	
		Women	Men	Women	Men
**Total**		16.3	76.0	5.2	17.5
**Age group^1^**					
15 – 29 years		0.1	0.1	0.0	0.0
30 – 39 years		0.9	2.3	0.1	0.4
40 – 49 years		3.7	12.5	1.1	2.6
50 – 59 years		9.0	46.7	3.4	11.3
60 – 69 years		21.3	125.8	8.2	31.8
70 – 79 years		42.4	224.9	13.9	52.5
80 years or older		76.0	270.2	20.7	55.5
**Area-level socioeconomic deprivation^1^**					
Low	Quintile 1	15.8	59.8	4.1	13.3
Medium	Quintile 2	17.2	66.4	4.6	14.1
	Quintile 3	16.6	66.2	6.1	14.3
	Quintile 4	19.4	76.6	6.2	18.3
High	Quintile 5	21.3	84.7	7.7	22.4

^**1**^Age-standardised values calculated using the age groups 15 – 19 years to 80 – 84 years in five-year intervals and older than 85 years using the population of Germany as of December 31, 2022 as a reference; the first quintile corresponds to districts with low socioeconomic deprivation, quintiles 2 – 4 correspond to districts with medium socioeconomic deprivation, the fifth quintile corresponds to districts with high socioeconomic deprivation.
