# Corrigendum: Diabetes-related amputations in Germany: analysis of time trend from 2015 to 2022 and differences by area-level socioeconomic deprivation

**DOI:** 10.25646/12869

**Published:** 2024-10-17

**Authors:** Oktay Tuncer, Yong Du, Niels Michalski, Lukas Reitzle

**Affiliations:** Robert Koch Institute, Department of Epidemiology and Health Monitoring Berlin, Germany

**Authors:** Oktay Tuncer, Yong Du, Niels Michalski, Lukas Reitzle

**Institution:** Robert Koch Institute, Department of Epidemiology and Health Monitoring, Berlin, Germany

J Health Monit. 2024;9(2):e12026. doi: 10.25646/12026

In Section 2, ‘Indicator’, on page 2, the inclusion criterion for hospital cases with diabetes was not fully reproduced; two ICD-10-GM codes (E11.- and E13.-) were missing.

The correct sentence is: ‘The analysis included all hospital cases with a main or secondary diagnosis of diabetes (ICD-10-GM codes E10.-/E11.-/E13.-/E14.-) of persons aged 15 years and older in the years 2015–2022 for whom a lower limb amputation was documented during hospitalisation.’

The article has been corrected accordingly.

